# Vascular Shutdown by Photodynamic Therapy Using Talaporfin Sodium

**DOI:** 10.3390/cancers12092369

**Published:** 2020-08-21

**Authors:** Taketo Suzuki, Mamoru Tanaka, Makiko Sasaki, Hiroshi Ichikawa, Hirotada Nishie, Hiromi Kataoka

**Affiliations:** Department of Gastroenterology and Metabolism, Nagoya City University Graduate School of Medical, Nagoya 467-8601, Japan; bellrinn3@yahoo.co.jp (T.S.); makiko@med.nagoya-cu.ac.jp (M.S.); piposikidaisuki@gmail.com (H.I.); nishix589998@gmail.com (H.N.); hkataoka@med.nagoya-cu.ac.jp (H.K.)

**Keywords:** photodynamic therapy, Rho-GTP pathway, talaporfin sodium, vascular shutdown effect

## Abstract

Photodynamic therapy (PDT) is an attractive cancer treatment modality. Talaporfin sodium, a second-generation photosensitizer, results in lower systemic toxicity and relatively better selective tumor destruction than first-generation photosensitizers. However, the mechanism through which PDT induces vascular shutdown is unclear. In this study, the in vitro effects of talaporfin sodium-based PDT on human umbilical vein endothelial cells (HUVECs) were determined through cell viability and endothelial tube formation assays, and evaluation of the tubulin and F-actin dynamics and myosin light chain (MLC) phosphorylation. Additionally, the effects on tumor blood flow and tumor vessel destruction were assessed in vivo. In the HUVECs, talaporfin sodium-based PDT induced endothelial tube destruction and microtubule depolymerization, triggering the formation of F-actin stress fibers and a significant increase in MLC phosphorylation. However, pretreatment with the Rho-associated protein kinase (ROCK) inhibitor, Y27632, completely prevented PDT-induced stress fiber formation and MLC phosphorylation. The in vivo analysis and pathological examination revealed that the PDT had significantly decreased the tumor blood flow and the active area of the tumor vessel. We concluded that talaporfin sodium-based PDT induces the shutdown of existing tumor vessels via the RhoA/ROCK pathway by activating the Rho-GTP pathway and decreasing the tumor blood flow.

## 1. Introduction 

Photodynamic therapy (PDT), an approved and increasingly popular cancer treatment modality, involves the administration of a photosensitizing chemical substance (the photosensitizer) together with visible light irradiation of a specific wavelength to a tumor. The photochemical reaction between the accumulated photosensitizer molecules and light leads to the production of highly potent reactive oxygen species that kill the tumor cells directly [1,2,3]. PDT has the advantages of being able to destroy tumors selectively and eliciting fewer side effects [2]. The minimization of toxicity to the target tissue results from the low dark toxicity and marginal selectivity of the photosensitizer, the spatial confinement of the activating light, and the short lifetimes of (and hence short distances travelled by) the toxic reactive molecule species that are generated in response to the light-based activation of the photosensitizer [4]. Thus, PDT has been widely used for the treatment of various malignancies, such as esophageal, gastric, lung, breast, head and neck, bladder, and prostate tumors [1]. It has been observed that, compared with other therapies, PDT of tumors often results in higher cure and lower recurrence rates in patients [5].

Aside from its localized destruction of tumors by direct killing of the tumor cells, PDT also induces immunological effects [4,6,7]. We have previously reported that PDT could induce immunogenic cell death with immunogenicity through the expression of calreticulin and the release of high-mobility group box 1 protein (HMGB1) in a mouse model [3]. Moreover, it has been reported that PDT can rapidly damage the microvasculature of tumors [8,9,10]. Such PDT-induced damage to the microvasculature results in a significant decrease of the blood flow and severe tissue hypoxia [11].

The tumor vasculature is an attractive therapeutic target because the vessels supply oxygen and nutrients that support the survival of tumor cells and are the main routes for tumor metastasis [12]. The aim of antivascular therapy using vascular-disrupting agents (VDAs) is the destruction of the established tumor vasculature, which will result in the death of secondary tumor cells with the rapid and extensive decrease of the tumor blood flow [13]. One such VDA is combretastatin A4 3ʹ-O-phosphate (CA4P), which is being tested in clinical trials [14]. CA4P can induce the depolymerization of interphase microtubules and contractility of actinomyosin and reorganize the actin cytoskeleton through activation of the RhoA/Rho-associated protein kinase (ROCK) pathway [15].

However, there is a limitation of PDT. Although the first-generation photosensitizer porfimer sodium (Photofrin^®^) has been widely used in the clinical setting, it results in long-term skin photosensitivity and its short wavelength of absorption limits tissue penetration [2,16]. By contrast, talaporfin sodium (TS; mono-l-aspartyl chlorin e6, NPe6, Laserphyrin^®^), a second-generation photosensitizer, requires a shorter sunshade period (2 weeks) than the first-generation photosensitizers (4 weeks) because it is rapidly cleared from the body. Furthermore, the excitation wavelength of the diode laser that is used with TS is longer (664 nm) than that of the excimer dye laser (630 nm) that is used with porfimer sodium, which means that the antitumor effect of TS reaches deeper than the submucosal layer, including even the muscular layer [17]. A recent phase II study concluded that PDT using TS (hereinafter, TS-PDT) is a safe and curative salvage treatment for local failure following chemoradiotherapy or radiotherapy alone in patients with esophageal cancer [18]. 

The vascular shutdown effect is commonly known to be photosensitizer dependent [19]. The selective vascular effects of TS have been demonstrated in preclinical studies [20,21,22,23,24]. Compared with porfimer-based PDT, TS-PDT resulted in a more significant vascular shutdown effect and consequently greater tumor necrosis resulting from ischemia [25]. In clinical trials, TS-PDT has been used for the management of bleeding after cold snaring in patients with esophageal cancer. However, the mechanism through which PDT promotes vascular shutdown remains unclear. We hypothesized that TS-PDT could modulate pericyte contractility and cause changes in the microstructure and stability of the tumor vasculature. In this study, we have determined that the shutdown of existing tumor vessels by TS-PDT involves the RhoA/ROCK pathway.

## 2. Results

### 2.1. TS-PDT Induced the Death of Vein Endothelial Cells 

**Figure 1:**
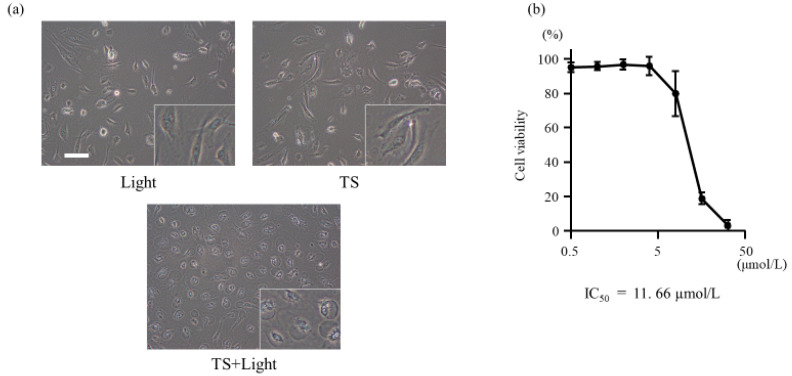
Effect of photodynamic therapy using talaporfin sodium (TS-PDT) on the viability of human umbilical vein endothelial cells (HUVECs). (**a**) HUVECs were treated with light irradiation alone, TS alone, or TS-PDT as indicated. Phase-contrast microscopy images were obtained (original magnification, 200×; scale bar, 50 µm). (**b**) Changes in the viability of the cells post TS-PDT were measured through cell proliferation assays. The IC_50_ value was calculated. Data are presented as the means ± SE of three independent experiments.

The cell proliferation assay was used to evaluate whether TS-PDT could induce the death of vein endothelial cells. As shown in Figure 1b, TS-PDT induced the death of human umbilical vein endothelial cells (HUVECs) in a dose-dependent manner, with a half-maximal inhibitory concentration of 11.66 µmol/L. However, there was no significant toxicity induced in the HUVECs by the administration of TS alone (i.e., in the absence of light) (Figure S1). The same results were obtained with the MKN45, HT29, and HCT116 cell lines [26,27].

### 2.2. TS-PDT Induced the Destruction of Endothelial Tubes 

**Figure 2:**
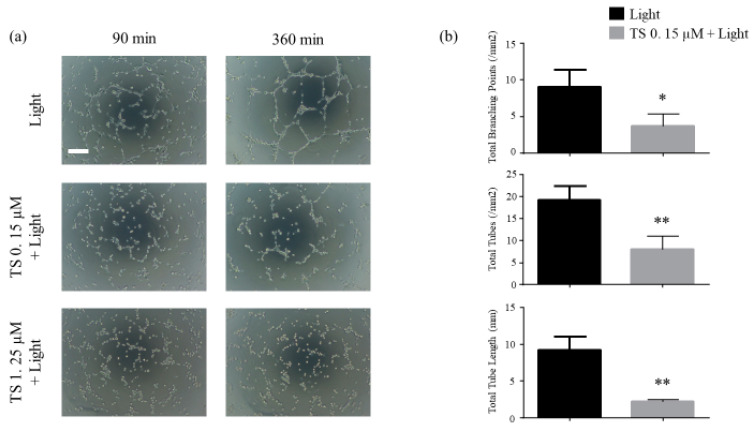
Photodynamic therapy using talaporfin sodium (TS-PDT) inhibited endothelial tube formation in human umbilical vein endothelial cells (HUVECs). Tube disruption was determined using the Endothelial Tube Formation Assay Kit. After HUVECs were seeded on the Matrigel, endothelial tubes formed after 6 h and were treated as indicated. After 6 h of treatment, the following were evaluated: (**a**) Tube disruption was quantified 6 h after treatment with light irradiation alone or TS-PDT. (original magnification, 100×; scale bar 100 µm). (**b**) The total tube length, total number of tubes, and total number of branching points were evaluated. Data from the bar graphs are presented as the mean ± SEM from four independent experiments. The results were analyzed using Welch’s *t*-test; * *p* < 0.05 and ** *p* < 0.01 compared with the control group.

Using the endothelial tube formation assay, we investigated whether TS-PDT could destroy the endothelial tubes. The results revealed that destruction of the tubes was initiated at approximately 90 min post TS-PDT and was almost completed within 300 min. TS-PDT significantly reduced the total tube lengths, total number of tubes, and total number of branching points of the tubes (Figure 2).

### 2.3. TS-PDT Triggered Depolymerization of the Microtubules and the Formation of F-Actin Stress Fibers 

**Figure 3:**
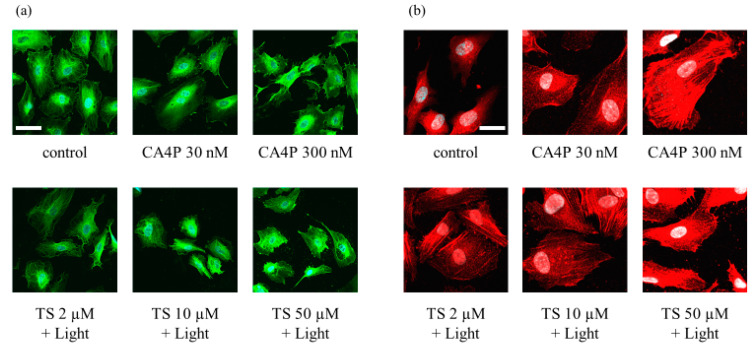
Photodynamic therapy using talaporfin sodium (TS-PDT) caused depolymerization of the microtubules and the formation of F-actin stress fibers in human umbilical vein endothelial cells (HUVECs), as shown by immunostaining and confocal microscopy. HUVECs were subjected to TS-PDT or pretreated with combretastatin A4 3-O-phosphate (CA4P) for 1 h, as indicated. (**a**) The microtubules were stained with an anti-tubulin antibody (green) and the nuclei were visualized (blue). (**b**) Cell-spanning F-actin stress fibers were stained using an anti-F-actin antibody (red). One representative experiment out of three is shown here (original magnification, 400×; scale bar, 20 µm).

To further evaluate the changes in vascular endothelial cells in response to TS-PDT, we observed the effects of the therapy on components of the cytoskeleton; namely, tubulin and actin filaments. The TS-PDT induction of microtubule depolymerization and actin stress fiber formation in HUVECS was examined in vitro through immunostaining. As a positive control, cells were incubated with CA4P. TS-PDT and CA4P individually induced depolymerization of the microtubules in a dose-dependent manner (Figure 3a). Individually, TS-PDT and CA4P also triggered the formation of thick F-actin stress fibers that spanned the cell (Figure 3b).

### 2.4. TS-PDT Activated the Myosin Light Chain 

**Figure 4:**
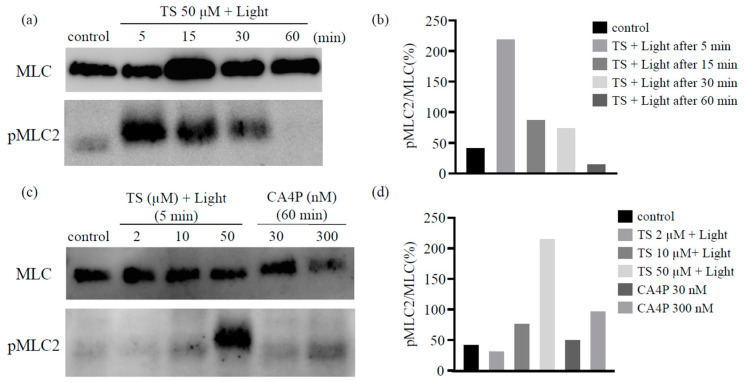
Photodynamic therapy using talaporfin sodium (TS-PDT) causes phosphorylation of the myosin light chain (MLC) in human umbilical vein endothelial cells. The TS-PDT induction of MLC phosphorylation was investigated in vitro using western blot analysis. (**a**,**b**) MLC was used as a loading control and MLC phosphorylation was measured at various time points. (**a**) Western blot analysis and (**b**) quantification of the western blot bands. (**c**,**d**) MLC phosphorylation at 5 min post TS-PDT of different doses was measured and compared with that observed using CA4P. (**c**) Western blot analysis and (**d**) quantification of the western blot bands. One representative experiment out of three is shown here. The Whole Blots for Western Blot analysis for Figure 4a,c are shown in Figure S2a,b.

It has been reported that the disassembly of the microtubules upon CA4P treatment could lead to the activation of the RhoA pathway, which would affect actin remodeling. Therefore, we used western blot analysis to determine the levels of the phosphorylated form of the myosin light chain (MLC), a Rho-kinase downstream effector, following TS-PDT in vitro. The phosphorylation of MLC was observed to have increased significantly within 5 min post TS-PDT and continued until 30 min (Figure 4a,b). Moreover, the extent of MLC phosphorylation induced by TS-PDT was higher than that induced by CA4P (Figure 4c,d). 

### 2.5. The Effects of TS-PDT are Triggered via the RhoA/Rho-Associated Protein Kinase/Myosin Light Chain Pathway 

**Figure 5:**
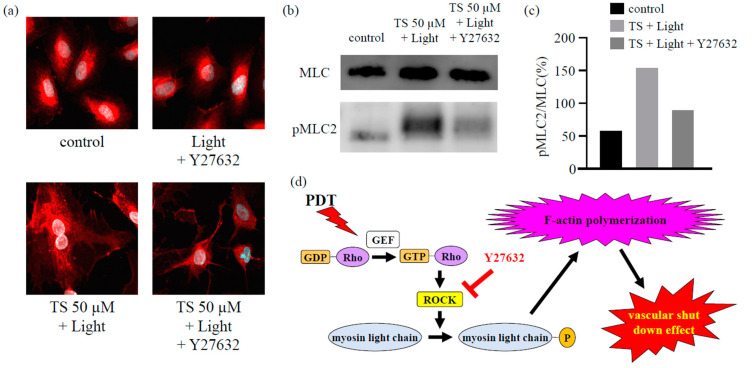
The inhibition of Rho-associated protein kinase (ROCK) inhibited the formation of actin stress fibers and the phosphorylation of myosin light chains (MLCs) in human umbilical vein endothelial cells subjected to photodynamic therapy using talaporfin sodium (TS-PDT). The inhibition of (**a**) actin stress fiber formation and (**b**,**c**) MLC phosphorylation upon treatment with the ROCK inhibitor Y27632 was investigated using (**a**) immunostaining (original magnification, 400×; scale bar, 20 µm), (**b**) western blot analysis, and (**c**) quantification of the western blot bands. One representative experiment out of three is shown here. The Whole Blots for Western Blot analysis for Figure 5b are shown in Figure S2c. (**d**) Mechanism through which TS-PDT exerts its vascular shutdown effect via the Rho-GTP pathway.

As described in Section 2.3 and Section 2.4, immunostaining (Figure 3) and western blot analysis (Figure 4) revealed that TS-PDT could activate the target downstream of RhoA for the formation of stress fibers and MLC. However, treatment with the ROCK inhibitor Y27632 completely prevented both the TS-PDT-induced formation of stress fibers and phosphorylation of MLC in the HUVECs (Figure 5a–c). These data suggested that the effects of TS-PDT were mainly mediated through the RhoA/ROCK pathway (Figure 5d).

### 2.6. TS-PDT Reduced Tumor Blood Flow in a Mouse Model of Subcutaneous Tumor

We also attempted to evaluate whether vascular shutdown occurs in vivo in response to TS-PDT. Mice with xenografted subcutaneous tumors were established using the HCT116 colon cancer cell line. At 2 h after the intravenous injection of TS, the tumors were irradiated using a diode laser system (664 nm). The blood flow in and around the tumor was measured using OMEGAZONE2, a laser speckle blood flow (LSBF) analysis system. The serial changes of the flow pattern were compared against those of the control or the positive control (CA4P).

**Figure 6:**
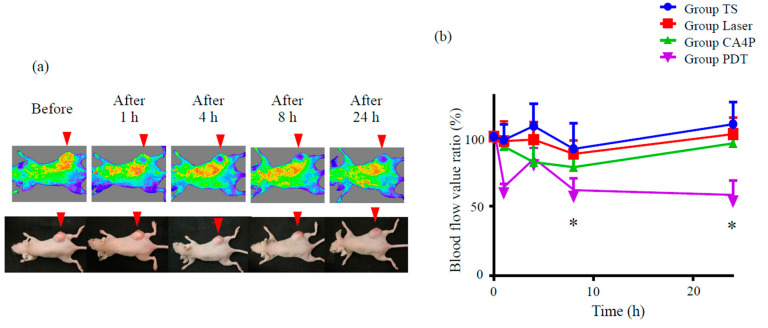
Photodynamic therapy using talaporfin sodium (TS-PDT) decreased tumor blood flow in a mouse model of HCT116 subcutaneous tumor. Mouse xenograft tumor models were established using the HCT116 colon cancer cell line. (**a**) Tumor blood flow was measured using a laser speckle blood flow analysis system, OMEGAZONE2. One representative experiment out of seven is shown here. (**b**) The blood flow ratio was calculated by dividing the flow post TS-PDT with the flow before TS-PDT. Data are presented as the means ± SE from seven independent experiments. The results for the different groups were analyzed and compared using the Bonferroni–Holm method; * *p* < 0.05.

No changes in tumor blood flow were observed in mice treated either with laser irradiation only (without photosensitizer administration) or TS only (administration of TS, followed by housing in the dark) (Figure S3). CA4P slightly reduced tumor blood flow compared to these control groups. TS-PDT was observed to be more potent than CA4P in reducing tumor blood flow (*p* ≤ 0.05, Figure S3, Figure 6). The blood flow improved temporarily at 4 h after TS-PDT, which was likely due to the effect exerted by reactive oxygen species.

### 2.7. The TS-PDT-Induced Destruction of Tumor Vessels Was Verified Pathologically

**Figure 7:**
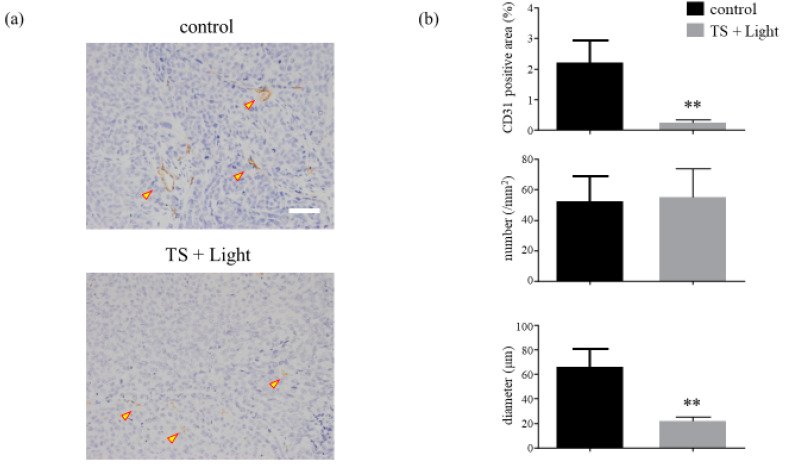
Analysis of the microvessel density of HCT116 xenograft tumors treated with photodynamic therapy using talaporfin sodium (TS-PDT). (**a**) Histological sections of the control (laser-treated only) and TS-PDT-treated HCT116 tumors were stained for the detection of CD31 (original magnification, 200×; scale bar, 50 µm). One representative experiment out of five is shown here. (**b**) The mean area positive for CD31 (%), number of microvessels/mm^2^, and diameter of the microvessel (µm) in the viable tumors were determined through morphometric image analysis. Data are presented as the means ± SE from five independent experiments. The results were analyzed using Welch’s *t*-test; * *p* < 0.05 and ** *p* < 0.01.

In addition to the evaluation using OMEGAZONE2, the tumors were excised from the various mouse groups and evaluated pathologically by immunohistochemical staining for CD31 (a marker for vascular endothelial cells) at 24 h after TS-PDT. It was found that TS-PDT led to significant reductions in the active areas and diameters of the tumor vessels compared with those of the control, although the number of active areas remained unchanged (Figure 7, Figure S4).

## 3. Discussion

Vascular shutdown as an effect of PDT has attracted increasing attention of late. Therefore, to gain a better understanding of the underlying mechanisms involved, we attempted to identify the continuous changes in hemokinesis in the vessels during or after TS-PDT. Angiogenesis is a physiological process involving the growth of new blood vessels from preexisting ones and its role in cancer development has been well acknowledged [28]. If a tumor mass grows beyond a few millimeters in size, the occurrence of an “angiogenic switch” is expected [29]. The tumor vasculature is considered to be an important target for cancer therapy, and second-generation photosensitizers are taking advantage of the need of tumor cells to depend on a functional blood supply for continued growth [30]. In particular, vascular-targeted PDT has been developed as a treatment option against localized prostate cancer, where it can selectively ablate the prostate with minimal collateral damage to other organ structures. It has reported that vascular-targeted PDT using verteporfin and TOOKAD could effectively destroy tumors by inducing vessel endothelial cell injury [31,32,33,34], thereby disrupting vascular function [30,34,35]. PDT with verteporfin was observed to safely reduce the risk of vision loss in patients with subfoveal occult without classic choroidal neovascularization resulting from age-related macular degeneration and polypoidal choroidal vasculopathy [36]. Multicenter clinical trials have confirmed that vascular-targeted PDT is clinically efficacious and has an excellent safety profile [19].

In this study, we have demonstrated using HUVECs that TS-PDT induced cell death and the destruction of endothelial tubes in the cells in vitro (Figure 1 and Figure 2). Additionally, TS-PDT induced the depolymerization of tubulin and the polymerization of F-actin via destruction of the microtubules in the HUVECs (Figure 3). 

The shape and function of endothelial cells are maintained by the cytoskeleton, a key target for tubulin-binding VDAs [15]. CA4P is a VDA being developed for the clinical treatment of ovarian and other cancers [37]. It is active in isolated tumor perfusions in the absence of blood, which supports the theory that the event initiating vascular shutdown occurs directly in the endothelial cells and/or directly impacts vasoconstriction of the supplying arterioles. However, a more pronounced vascular effect was observed in tumors in vivo, implicating the additional role of blood cells [12]. Kretzschmann et al. reported that the disassembly of the microtubules upon treatment with CA4P could activate the RhoA pathway, which contributes to actin remodeling [38,39].

We found that TS-PDT could also trigger activation of the RhoA pathway. Additionally, a significant increase in the phosphorylation of MLC (a Rho-kinase downstream effector) was detected at 5, 15, and 30 min post TS-PDT administration (Figure 4). Several studies have demonstrated the importance of the RhoA/ROCK pathway in pericyte contractility and activation [24,40]. Y27632, a synthetic pyridine derivative that inhibits ROCK by competing with ATP for the active site of the kinase, was able to impair the TS-PDT-induced phosphorylation of MLC (Figure 5a,b). This indicates that ROCK acts downstream of RhoA and mediates the phosphorylation of MLC induced by TS-PDT, similar to the processes observed with CA4P. Therefore, we have confirmed that TS-PDT induces activation of the RhoA/ROCK pathway, which subsequently leads to MLC phosphorylation and increased actin polymerization and filament stabilization, which further drive stress fiber and focal adhesion assembly (Figure 5c).

Recently, a chick chorioallantoic membrane model was used as an in vivo assay for studying the application of PDT for eye diseases [41,42,43,44]. In our study, the vascular shutdown effect of TS-PDT was investigated for the first time in vivo using an LSBF analysis system (Figure 6). This analytical system conducts blood flow measurements using laser speckle imaging and has the following advantages: (1) blood flow is visualized as a two-dimensional image; (2) the area of blood flow measurement can be changed in saved image data; and (3) the averaged data do not consist of absolute values and can be used quantitatively in comparative studies [45]. Moreover, by using the LSBF imager, it was possible to link the vascular shutdown to the extent of the vascular response (as found with CD31) (Figure 7). Importantly, significant vascular shutdown was observed after TS-PDT with a relatively low dose of light (15 J/cm^2^). Several PDT studies have commonly used light energy doses of over 100 J/cm^2^ for killing carcinoma xenografts [46,47,48,49]. Our results therefore indicate that the light energy dose required for inducing vascular shutdown is much lower than that required to induce cancer cell death.

There are generally two distinct groups of vascular-targeted therapies: antiangiogenic agents and VDAs [50]. The TS-PDT applied in our study reduced the active area and diameter of the tumor vessels without changing their numbers (Figure 7), implying that TS-PDT is not an antiangiogenic agent but a vascular-disrupting approach.

## 4. Materials and Methods

### 4.1. Compounds 

CA4P (No. C3009) was procured from TCI (Tokyo, Japan). The ROCK inhibitor Y27632 was purchased from MERCK (Darmstadt, Germany). TS (mono-l-aspartyl chlorin e6, Laserphyrin^®^, Tokyo, Japan) was purchased from Meiji Seika (Tokyo, Japan).

### 4.2. Cell Culture 

The HUVECs (Cascade Biologics, Portland, OR, USA) were cultured in Dulbecco’s modified Eagle’s medium/F12 medium containing 10% fetal bovine serum (FBS) and 10 ng/mL basic fibroblast growth factor (DS Pharma Biomedical Co. Osaka, Japan). The HCT116 human colon cancer cells (No. CCL-247, American Type Culture Collection, Manassas, VA, USA) were cultured in McCoy’s 5A medium (Sigma-Aldrich, St. Louis, MO, USA) containing 10% FBS and 1% ampicillin-streptomycin. All the cells were cultured under an atmosphere of 5% CO_2_ at 37 °C.

### 4.3. Photodynamic Therapy In Vitro

HUVECs were incubated for 24 h in the culture medium, following which they were incubated in culture medium supplemented with TS at varying concentrations (0.5–64 µM) for 4 h. The cells were washed once with phosphate-buffered saline (PBS), resuspended in the same buffer, and then irradiated using a light-emitting diode system (OptoCode Corporation, Tokyo, Japan) at a light energy dose of 16 J/cm^2^ (irradiance: 36 mW/cm^2^ × 444 s) and wavelength of 660 nm. Then, the PBS was replaced with culture medium and the cells were further incubated for a specified time prior to their analysis.

### 4.4. Cell Viability Assay 

HUVECs were seeded into 96-well culture plates at a density of 5 × 10^3^ cells/well in a 100 μL volume and incubated overnight. The cells were then subjected to PDT with various doses of TS in vitro and incubated further for 24 h. Cell viability was investigated with Cell Counting Kit-8 (Donjindo Laboratories, Kumamoto, Japan) and a microplate reader (SPECTRA MAX340; Molecular Devices, Silicon Valley, CA, USA). The survival rate was expressed as the percentage of live cells relative to untreated control cells, and the half-maximal (50%) inhibitory concentration (IC_50_) was calculated from the survival curve. Data are expressed as the means ± SE from three independent experiments.

### 4.5. Endothelial Tube Formation Assay 

Tube disruption was measured using the Endothelial Tube Formation Assay Kit (Cell Biolabs, Inc., San Diego, CA, USA). The bottom of each well of a cell culture plate (96-well) was coated with a thin layer of ECM gel (50 μL/well; polymerization time: 60 min at 37 °C). HUVECs (1 × 10^4^ cells in a 50 μL volume) were then layered over the solidified ECM gel and incubated for 1 h at 37 °C. Thereafter, the cells were subjected to TS-PDT in vitro and incubated for 6 h at 37 °C. The total tube length, total number of tubes, and total number of branching points of the tubes were counted using ImageJ software. The resultant data, expressed as the mean ± SEM from four independent experiments, were compared with the results from the control group.

### 4.6. Immunocytochemistry and Confocal Laser Scanning Fluorescence Microscopy 

HUVECs were seeded onto 8-well glass slides (Lab-Tek, MERCK, Darmstadt, Germany) at a density of 1 × 10^5^ cells per well, incubated for 24 h, and then subjected to TS-PDT in vitro. Thereafter, the cells were fixed with paraformaldehyde and incubated with primary antibodies against tubulin (ab18251, Abcam, Cambridge, MA, USA) or F-actin (bs-1571R, Bioss, Woburn, MA, USA). Alexa Fluor 488-conjugated goat anti-rabbit IgG (H + L; Invitrogen, Tokyo, Japan) was used as the secondary antibody. All sections were counterstained using 4′,6-diamidino-2-phenylindole (DAPI; Kirkegaard and Perry Laboratories, Gaithersburg, MD, USA). Images were obtained using a confocal laser scanning fluorescence microscope (FV3000; Olympus, Tokyo, Japan). After incubating the HUVECs for 24 h, the medium was replaced with medium supplemented with CA4P (30–300 nM) and incubation was carried out for 1 h. Fluorescent immunostaining was then carried out as described above. Additionally, another set of HUVECs was preincubated with the ROCK inhibitor Y27632 (10 µM) before TS-PDT in vitro and fluorescent immunostaining. The results presented are from one representative experiment out of three experiments.

### 4.7. Western Blot Analysis 

Following TS-PDT of the HUVECs, the intracellular proteins were extracted from the cells using a cell lysis buffer (Cell Signaling Technology, Beverly, MA, USA). The concentration of the total proteins was measured using a protein assay kit (Bio-Rad Laboratories, Hercules, CA, USA). The protein samples were then separated on Long-Life Tris-Glycine eXtended (TGX) gels (Bio-Rad) and the protein bands were transferred onto a nitrocellulose membrane (Schleicher and Schuell BioScience, Dassel, Germany). The membrane was incubated with the primary anti-pMLC antibody (1:1000 dilution; #3671; Cell Signaling Technology, Danvers, MA, USA) after blocking. Thereafter, the membrane was washed extensively and then incubated with the secondary antibody. The signals were quantified using the ECL Plus Western Blotting Detection System (GE Healthcare, Tokyo, Japan), Image Quant LAS 4000 Mini System (GE Healthcare), and ImageJ software. The membranes were probed with the anti-MLC2 antibody (1:1000 dilution; #8505; Abcam) as an internal control. HUVECs incubated with Y27632 before TS-PDT in vitro or with CA4P alone (30 or 300 nM) or Y27632 alone (10 µM) were similarly investigated using western blotting. The results presented are from one representative experiment out of three experiments.

### 4.8. Animals and Tumor Models 

Pathogen-free female nude mice (BALB/c Slc-nu/nu), aged 4 weeks and weighing 20–25 g, were purchased from Japan SLC, Inc. (Hamamatsu City, Shizuoka, Japan). Xenograft tumor models were established by implanting 100 μL of medium containing 1 × 10^6^ HCT116 cells subcutaneously in the right flank of each animal. The use of animals and the procedures employed in this study were approved by the Animal Research Committee of Nagoya City University (Project number: H29M-38).

### 4.9. Photodynamic Therapy In Vivo

At 20 days after inoculation of the HCT116 tumor cells, the xenograft-carrying mice were injected intravenously with 6.25 μmol/kg TS via the tail vein. After 2 h of TS treatment, the tumors were irradiated with light of 664 nm wavelength (OK Fiber Technology, Kyoto, Japan) at a dose 15 J/cm^2^ (irradiance: 150 mW/cm^2^ × 100 s). The temporal changes in tumor blood flow were monitored using an LSBF analysis system (OMEGAZONE2; OMEGAWAVE Inc., Tokyo, Japan). Data from the different groups are expressed as the means ± SE from seven independent experiments.

### 4.10. Immunohistochemistry 

The tumors on the mice were excised and immediately fixed in formalin and embedded in paraffin blocks. The block specimens were then sectioned (4 µm) and stained using an anti-CD31 monoclonal antibody (1:100 dilution; bs-5913R; Cell Signaling Technology). Random fields were captured under a microscope and the areas positive for CD31 were quantified using ImageJ software (NIH, Bethesda, MD, USA). Data are expressed as the mean ± SE from five independent experiments.

### 4.11. Statistical Analysis 

Descriptive statistical analysis was carried out using the statistical package GraphPad Prism 8 (GraphPad Software, Inc., San Diego, CA, USA). A *p*-value of less than 0.05 was considered statistically significant for all analyses.

## 5. Conclusions

We have demonstrated that TS-PDT induced vascular shutdown through activation of the RhoA/ROCK pathway, leading to the polymerization of F-actin in vitro. Additionally, TS-PDT significantly decreased blood flow in the tumor in vivo. On the basis of the applicability and characteristics of TS presented in this study, we surmise that it could be an important component responsible for the vascular shutdown resulting from the use of PDT, thereby contributing to the antitumor effects of this treatment modality.

## Supplementary Materials

The following are available online at https://www.mdpi.com/2072-6694/12/9/2369/s1, Figure S1: Changes in cell viability after treatment with the drug (without irradiation), Figure S2: Whole western blots (uncropped blots) showing all the bands with molecular weight markers, Figure S3: Laser speckle blood flow image showing the in vivo antivascular effects of TS-PDT on HCT116 xenografts in nude mice, Figure S4: Pathological expression of CD31 in vivo revealing the antivascular effects of TS-PDT.
