# Supplementary material for: Vascular Shutdown by Photodynamic Therapy Using Talaporfin Sodium
Source: Cancers (Basel). 2020 Aug 21;12(9):2369. doi: 10.3390/cancers12092369 (PMC7563359; doi:10.3390/cancers12092369)
Supplement: Supplementary file 1 [file cancers-12-02369-s001.pdf]

cancers

Supplementary Materials

Vascular Shutdown by Photodynamic Therapy Using Talaporfin Sodium

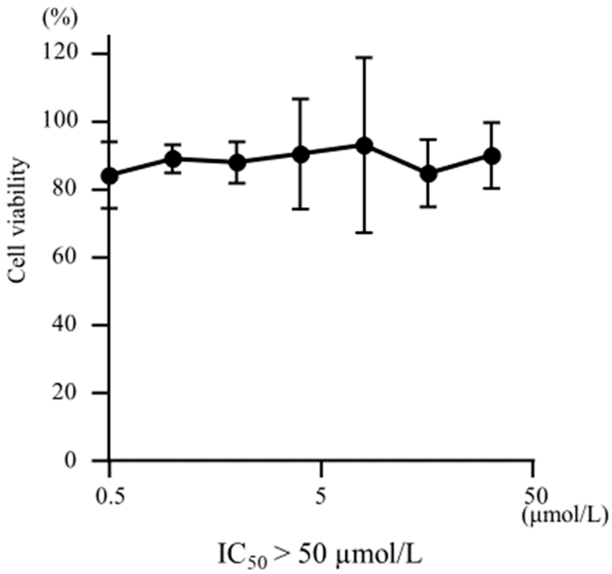

Figure S1. Changes in cell viability after treatment with the drug (without irradiation).

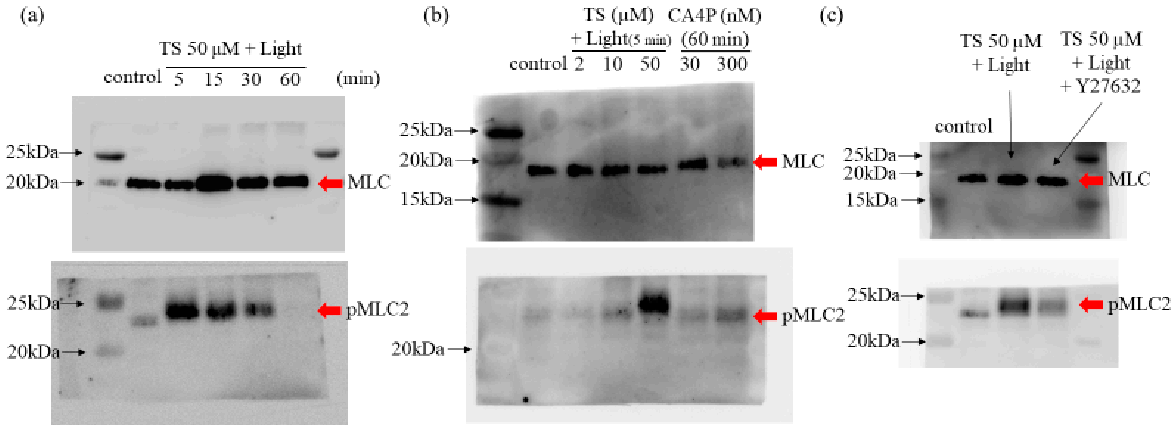

Figure S2. Whole western blots (uncropped blots) showing all the bands with molecular weight markers. (a), (b) and (c) belong to Figure 4a, Figure 4c, and Figure 5b.

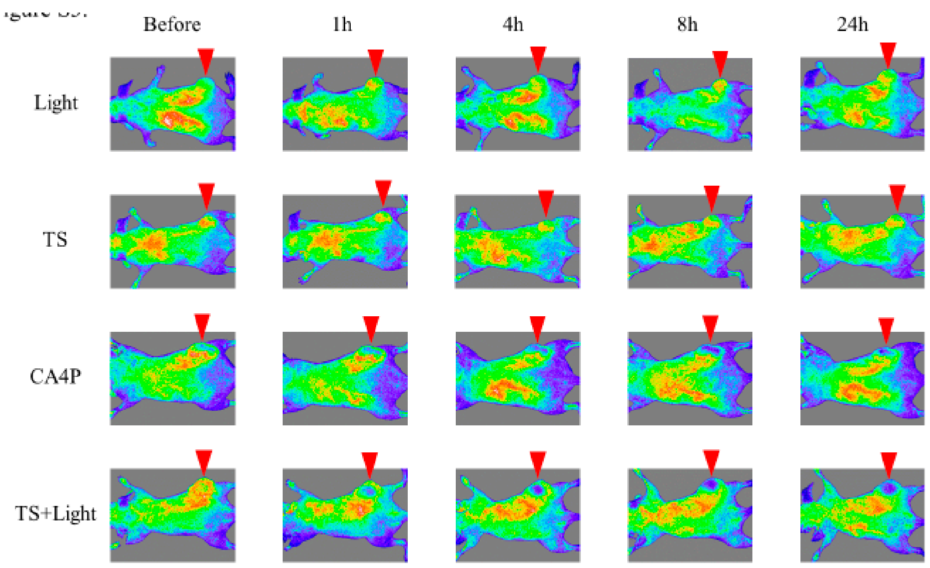

Figure S3. Laser speckle blood flow image showing the in vivo antivascular effects of TS-PDT on HCT116 xenografts in nude mice.

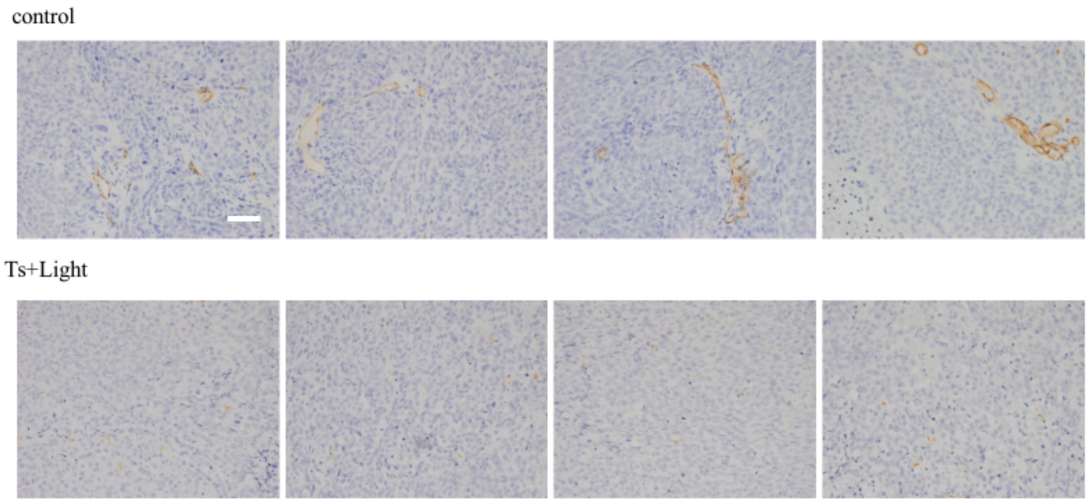

Figure S4. Pathological expression of CD31 in vivo revealing the antivascular effects of TS-PDT. (original magnification, 200×; scale bar, 50 μm)

© 2020 by the authors. Licensee MDPI, Basel, Switzerland. This article is an open access article distributed under the terms and conditions of the Creative Commons Attribution (CC BY) license (http://creativecommons.org/licenses/by/4.0/).
